# Mortality risk factors among hospitalized children with severe pertussis

**DOI:** 10.1186/s12879-021-06732-1

**Published:** 2021-10-12

**Authors:** Tingting Shi, Ling Wang, Shuling Du, Huifeng Fan, Minghua Yu, Tao Ding, Xuehua Xu, Dongwei Zhang, Li Huang, Gen Lu

**Affiliations:** 1 Department of Respiratory, Guangzhou Women and Children’s Medical Center, Guangzhou Medical University, No.9, Jinsui Road, Zhujiang New City, Tianhe District, Guangzhou, 510120 Guangdong China; 2 Department of Neonatology, Guangzhou Women and Children’s Medical Center, Guangzhou Medical University, Guangzhou, China; 3 Key Laboratory of Tropical Disease Control, Ministry of Education, Sun Yat-Sen University, Guangzhou, China; 4 Department of Cardiology, Guangzhou Women and Children’s Medical Center, Guangzhou Medical University, Guangzhou, China; 5 Pediatric Intensive Care Unit, Guangzhou Women and Children’s Medical Center, Guangzhou Medical University, Guangzhou, China

**Keywords:** Children, Pertussis, Pediatric intensive care unit, Risk factor

## Abstract

**Background:**

Some children hospitalized for severe pertussis need intensive care; moreover, some children die because of deterioration alone or in combination with other complications. The purpose of this study was to identify the mortality risk factors among hospitalized children with severe pertussis.

**Methods:**

This study evaluated the medical records of 144 hospitalized children with severe pertussis at the Guangzhou Women and Children’s Medical Centre between January 2016 and December 2019.

**Results:**

The median age of patients was 2 months (IQR 1–4 months), with 90.3% of the patients aged < 6 months and 56.9% of the patients aged < 3 months. A total of 38 patients were admitted to intensive care unit (ICU), 13 patients died, and the mortality of severe pertussis was 34.2%, with patients younger than 6 weeks accounting for 76.9% of the deaths. On the multivariate analysis, the independent risk factors for death were WBC > 70.0 × 10^9^/L (odds ratio [OR], 230.66; 95% confidence interval [CI], 5.16–10,319.09 P = 0.005) and pulmonary hypertension (PH) (OR 323.29; 95% CI 16.01–6529.42; P < 0.001).

**Conclusion:**

Severe pertussis mainly occurred in children aged < 3 months. The mortality of severe pertussis was 34.2%, with patients younger than 6 weeks accounting for the majority of the deaths. We recommend the first dose of diphtheria-tetanus-pertussis (DTP) should be advanced to the age of 2 months or even 6 weeks. The presence of a WBC > 70.0 × 10^9^/L and PH were the prognostic independent variables associated with death.

## Background

Pertussis is a highly contagious acute respiratory infection disease that is one of the main reasons of infectious disease-related deaths in children [1]. Since the spread of infant and childhood pertussis vaccination in the 1940s in the world, the incidence of pertussis has decreased more than 80% [2]. However, during the last two decades, pertussis infections have re-emerged worldwide. The WHO estimated that pertussis affects nearly 240,000,000 children aged < 5 years old each year and causes 160,700 deaths in this age, with the mortality of 4% [1, 3]. In a study of infants, the mortality was 70% and higher in infants younger than 6 weeks (84%) [4]. In China, 30,027 children were diagnosed with pertussis in 2019, and the morbidity of pertussis was lower than that in other countries (21.5/million) [5]. The mortality of pertussis in China is not very clear. This may be due to the limitations of the laboratory tests in China, and it does not reflect the actual incidence.

In this study, we analysed 144 hospitalized paediatric patients with pertussis, including 38 children with severe pertussis who were admitted to the paediatric intensive care unit (PICU) or neonatal intensive care unit (NICU). The purpose of this study was to identify the mortality risk factors in hospitalized patients with severe pertussis. This information may be beneficial to effectively prevent and to institute management strategies early for severe cases.

## Methods

### Study design

This study included 144 patients with pertussis who were admitted to Guangzhou Women and Children’s Medical Centre between January 2016 and December 2019. These patients had tested respiratory tract specimens and were found to be positive for Bordetella pertussis. The patients were selected by identifying nasopharyngeal secretions sample positive for *B. pertussis* in polymerase chain reaction (PCR) with the testing kit produced by Sheng Xiang Biotech Co., Ltd. China. All patients received an indirect immunofluorescence virus test of nasopharyngeal secretions sample during the acute phase. Blood and sputum cultures were used to identify bacterial infections, Mycoplasma pneumoniae or fungal infections. All patients also underwent a chest X-ray examination, and some of them underwent high-resolution tomography (HRCT) based on the extensive lesions found on the chest X-ray examination. Patients with incomplete clinical data were excluded from this study.

This study was approved by the Ethics Committee of Guangzhou Women and Children’s Medical Centre, Guangzhou Medical University.The study was performed according to the ethical guidelines of the Declaration of Helsinki (7th revision).

### Data collection

For all the patients, data on demographics; clinical features; contact history; comorbidities; prematurity, including gestational age and birth weight); vaccination history; time of hospital and PICU/NICU admissions; laboratory findings; microbiological and radiological findings; treatments, including type of medicine used, type of respiratory support and type of life support (continuous renal replacement therapy (CRRT),exchange blood transfusion(ET), inhalation of nitric oxide (NO),and extracorporeal membrane oxygenation (ECMO)); and outcomes were collected. Contact history was defined as a close contact who had a preceding cough. Vaccination history was obtained from the vaccination records for each child. Severe pertussis was defined on the criteria of severe pertussis in the American in 2013 [6]: Children from 0 to 18 years of age with laboratory confirmed (PCR and/or positive culture) pertussis were eligible for enrolment if they had a PICU stay of at least 24 h or died. Hyperleukocytosis was defined as WBC ≥ 50 × 10^9^/L. Pulmonary hypertension (PH) was defined on the basis of the diagnosis and treatment of PH of the European Society of Cardiology (ESC) and the European Respiratory Society (ERS) [7]. Our patients were considered to have PH based on the judgement of the echocardiography (ECHO) results. No patients underwent cardiac catheterization to judge the PH, because of their young ages and the severity of the illness. Septic shock was defined according to the International Paediatric Sepsis Consensus Conference criteria [8]. Acute Respiratory Distress Syndrome (ARDS) is defined by the standards of the Paediatric Acute Lung Injury Consensus Conference criteria [9].

### Statistical analysis

A total of 144 children diagnosed with pertussis in our hospital from 2016 to 2019 were included. Categorical data were presented as frequency with the corresponding percentage, and continuous data were showed as median with the interquartile range (IQR). The χ^2^ or Fisher exact test was used to determine the associations between the categorical variables and pertussis. To determine the independent contribution of each variable, multivariable logistic regression models were performed. A binary outcome variable was generated that coded for whether the child was a survivor or non-survivor. All analyses were completed using statistical software R Version 3.6.1, and the significance level of all tests was determined at P < 0.05.

## Results

### Demographics and baseline characteristics

**Fig. 1:**
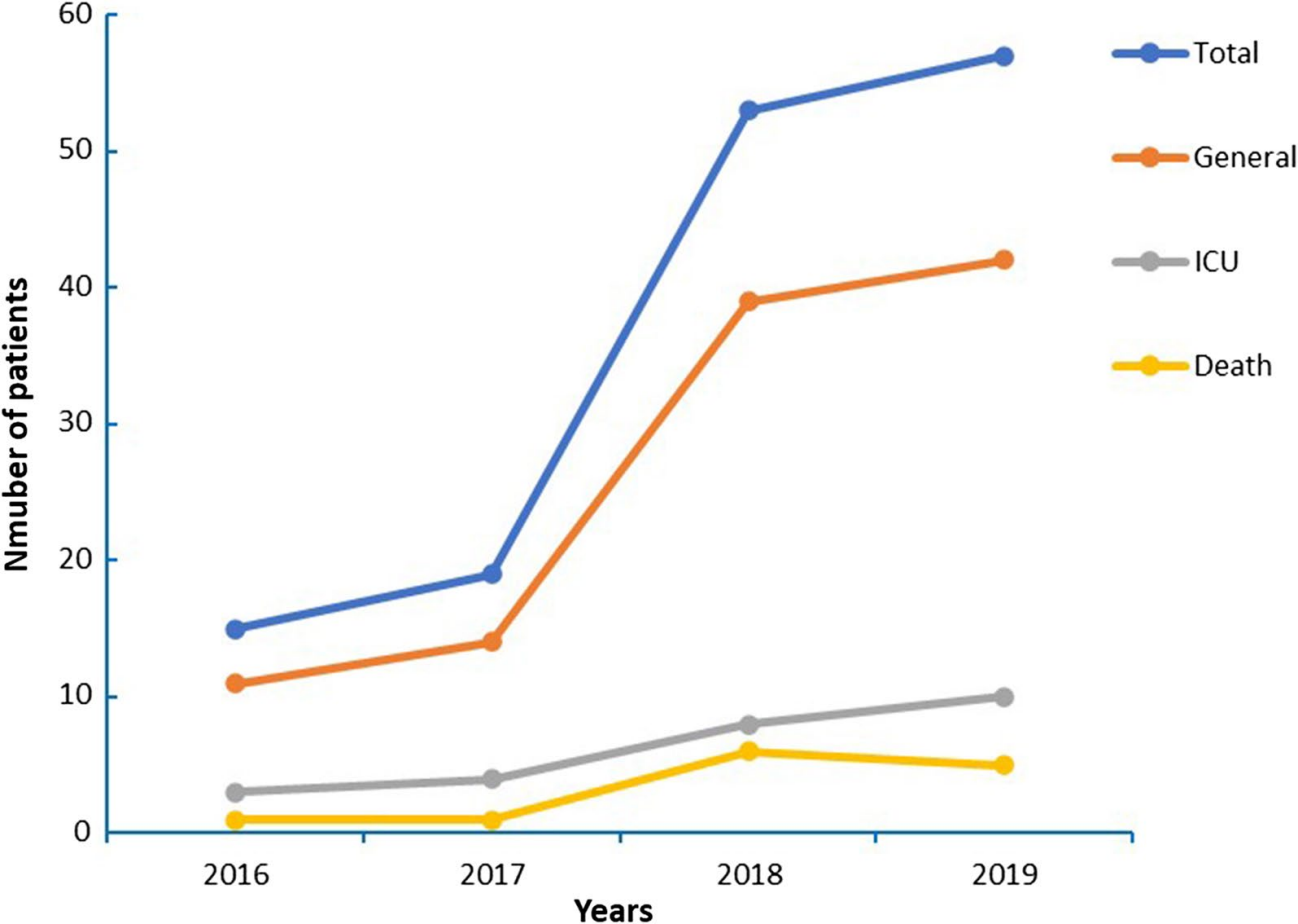
Distribution of the number of pertussis patients per year

In the 4-year period, 150 hospitalized children had a nasopharyngeal secretions sample positive for *B. pertussis*, including 38 patients admitted to the ICU and 13 patients who died. Six patients were excluded because of being discharged against medical advice. Of the 144 hospitalized patients enrolled, the demographics and baseline characteristics of children are presented in Table 1; 47.9% (69/144) were boys and 52.1% (75/144) were girls. The median age was 2 months (IQR 1–4 months), with ages ranging from 3 days to 4 years, in whom 90.3% (130/144) aged < 6 months and 56.9% (82/144) aged < 3 months, and most of the deaths were in patients < 3 months (92.3%, 12/13). In all, 11.8% (17/144) of patients had premature birth and 9.0% (13/144) of patients had low birth weight. A positive contact history was present in 72.2% (104/144) of patients. All the patients had pertussis vaccine records; 70.8% (102/144) of patients were unvaccinated for pertussis, with 56.9% (82/144) of the patients aged < 3 months, and 29.2% (42/144) patients had pertussis vaccination, including 18.8% (27/144) of patients receiving one dose, 6.9% (10/144) two doses and 3.5% (5/144) three doses. Of the 144 patients enrolled in the study, only eight patients had comorbid conditions, including chronic lung disease, abnormality in airways and neurologic disorders. Annual pertussis rates and annual pertussis deaths are shown in Fig. 1.

**Table 1:** Demographics and baseline characteristics of 144 hospitalized children with pertussis

Characteristics	Total	Survivors	Deaths	P-value
	N = 144	N = 131	N = 13	
	Number	Number	Number	
Demographics				
Male gender	69	63	6	1.000
Age distribution				< 0.001
< 6 weeks	28	18	10	
6 weeks to 2.9 months	54	52	2	
3 months to 5.9 months	48	47	1	
6 months to 11.9 months	10	10	0	
12 months to 3 years	2	2	0	
≥ 3 years	2	2	0	
Prematurity conditions	6	4	2	0.091
Gestational age				0.594
≥ 37 weeks	127	116	11	
32 weeks to 36.9 weeks	11	10	1	
28 weeks to 31.9 weeks	6	5	1	
< 28 weeks	0	0	0	
Birth weight				0.004
≥ 2500 g	131	122	9	
1500 g to 2499 g	10	8	2	
1000 g to 1499 g	2	0	2	
< 1000 g	1	1	0	
Positive contact history	104	92	12	0.112
Vaccination history				0.186
Any dose	102	89	13	
1 dose	27	27	0	
2 doses	10	10	0	
3 doses	5	5	0	
Underlying co-morbid conditions	8	7	1	0.540

### Clinical characteristics and complications

Cough (97.9%, 141/144) was the most common symptom in our study, and of these patients, 53.5% (77/144) had a whoop and 78.5% (113/144) had facial congestion, followed by cyanosis (45.1%, 65/144), fever (31.9%, 46/144) and shortness of breath (27.8%, 40/144). The median duration of cough was 13 days on admission (IQR, 7.25–20 days). The most common complication of pertussis was pneumonia (70.1%, 101/144), followed by respiratory failure (24.3%, 35/144), septic shock (10.4%, 15/144), pleural effusion (9.7%, 14/144) and PH (9.0%, 13/144). The abnormal clinical presentations, physical examinations and complications are showed in Table 2.

**Table 2:** Clinical characteristics and complications of 144 hospitalized children with pertussis

Characteristics	Total	Survivors	Deaths	P-value
	N = 144	N = 131	N = 13	
	Number	Number	Number	
Clinical symptoms				
Cough	141	130	11	0.022
Facial congestion	113	105	8	0.229
Whoop	77	67	10	0.088
Cough ≥ 14 days	71	70	1	0.002
Cyanosis	65	54	11	0.003
Fever	46	37	9	0.004
Axillary temperature ≥ 38.5℃	23	17	6	0.007
Fever ≥ 5 days	8	8	0	1.000
Shortness of breath	40	28	12	< 0.001
Fatigue with feeding	39	28	11	< 0.001
Vomiting/diarrhea	26	26	0	0.126
Altered sensorium	18	10	8	< 0.001
Seizures	17	12	5	0.008
Apnea	6	4	2	0.092
Physical exam findings				
Crackles	66	54	12	0.001
Wheezing	33	27	6	0.075
Oxygen saturation < 90%	31	20	11	< 0.001
Heart rate > 180/min(≤ 1 years) or > 160/min(> 1 year)	29	18	11	< 0.001
Respiratory rate > 70/min(≤ 1 years) or > 60/min(> 1 year)	15	5	10	< 0.001
Capillary refilling time(CRT) > 2 s	15	4	11	< 0.001
Decreased breath sounds	3	3	0	1.000
Complications				
Pneumonia	101	88	13	0.010
Respiratory failure	35	22	13	< 0.001
Septic shock	15	5	10	< 0.001
Pleural effusion	14	10	4	0.024
Pulmonary hypertension	13	1	12	< 0.001
Toxic encephalopathy	7	3	4	0.001
ARDS	6	0	6	< 0.001
Acute renal failure	6	1	5	< 0.001
Heart failure	4	1	3	0.002
Pneumorrhagia	3	1	2	0.022

### Laboratory, radiological and microbiological findings

**Fig. 2:**
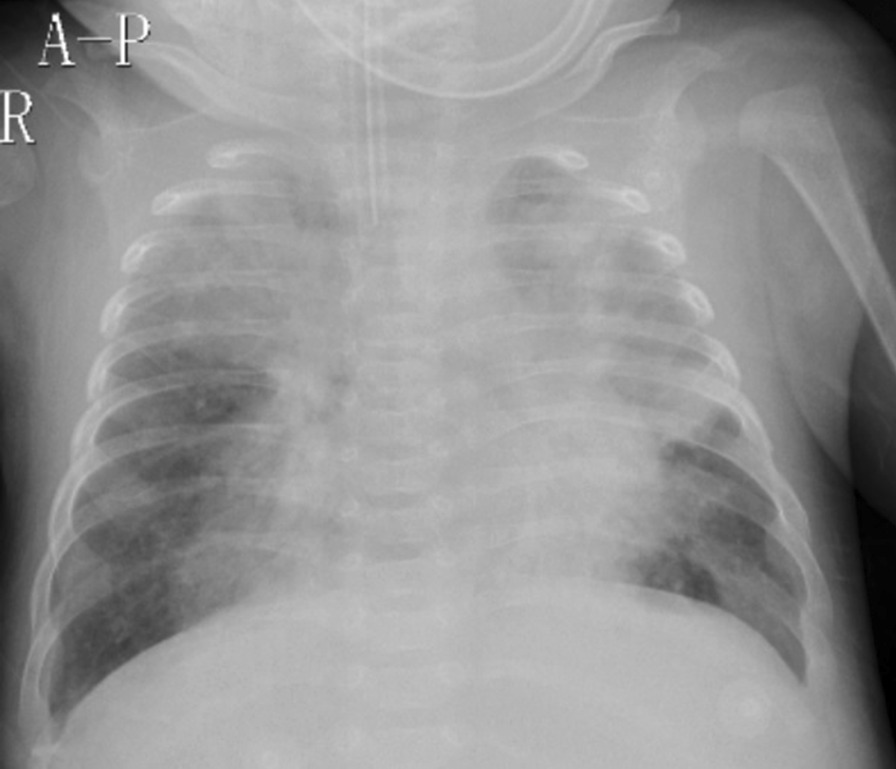
Chest radiograph showing bilateral diffuse infiltration and upper lungs are prominent indicative of acute respiratory distress syndrome

**Fig. 3:**
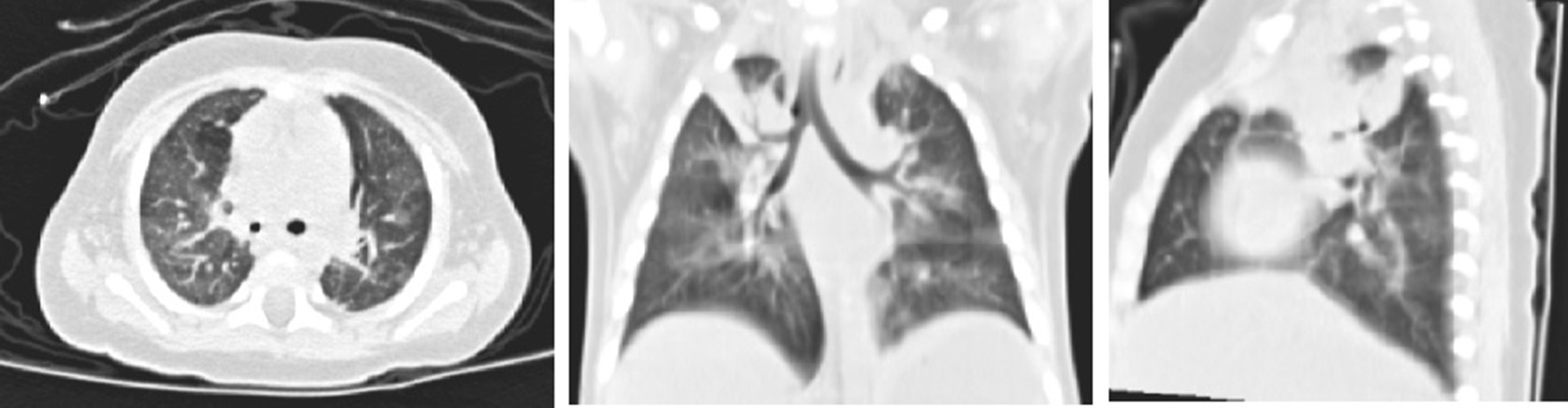
High-resolution CT scan of the chest revealing diffuse infiltration and areas of consolidation in the right upper in a 4-month-old girl

The abnormal laboratory, radiological and microbiological findings are showed in Table 3. Of the 144 patients, 79.2%(114/144) had varying degrees of increased white blood cell (WBC) counts and absolute lymphocyte counts, including 13.2% (19/144) having hyperleukocytosis (WBC > 50.0 × 10^9^/L), with 4.9% (7/144) of patients with WBC > 70.0 × 10^9^/L. Chest radiographic results mostly presented diffuse infiltration in both lungs. The patients died tended to show more severe infection (Fig. 2). Some severe patients presented severe consolidation on HRCT (Fig. 3). The other main radiographic finding was pleural effusion (10.4%, 15/144); 9.0% (13/144) of patients were considered to have PH based on the ECHO results, of which most were in the death group (92.3%, 12/13), and 46.2% (6/13) of the patients who died had severe PH. Among the 144 patients, besides *B. pertussis*, another causative agent was detected in 69.4% (100/144) of the cases, with other bacterial coinfections accounting for 26.4% (38/144), *M. pneumoniae* coinfections for 9.7% (14/144), *Chlamydia pneumoniae* coinfections for 3.5% (5/144), and viral coinfections for 29.9% (43/144). Of the bacterial coinfection cases, *Klebsiella pneumoniae* (6.9%, 10/144) and *Streptococcus pneumoniae* (6.9%, 10/144) were the most common typical bacteria isolated in patients with *B. pertussis* infection. The most common virus isolated were respiratory syncytial virus (16.0%, 23/144) and rhinovirus (6.9%, 10/144).

**Table 3:** The laboratory, radiological findings, and pathogenies of 144 hospitalized children with pertussis

Characteristics	Total	Survivors	Deaths	P-value
	N = 144	N = 131	N = 13	
	Number	Number	Number	
Laboratory index				
PO_2_ < 60 mmHg	32	27	5	0.260
PCO_2_ > 50 mmHg	35	28	7	0.024
Abnormal WBC				< 0.001
WBC < 30.0 × 10^9^/L	93	92	1	
WBC 30.0–50.0 × 10^9^/L	32	31	1	
WBC 50.0–70.0 × 10^9^/L	12	7	5	
WBC > 70.0 × 10^9^/L	7	1	6	
Absolute lymphocyte count > 20 × 10^9^/L	44	34	10	< 0.001
Hemoglobin < 80 g/L	14	10	4	0.024
C-reactive protein > 30 mg/L	34	26	8	0.002
Serum albumin < 35 g/L	27	21	6	0.023
Lactate dehydrogenase > 500 U/L	14	8	6	< 0.001
APTT > 50 s	9	4	5	< 0.001
Aspartate aminotransferase > 100 U/L	7	4	3	0.017
Creatine kinase-MB fraction > 100 U/L	4	3	1	0.318
Creatinine > 62 mg/d	3	0	3	0.001
Alanine aminotransferase > 100 U/L	2	1	1	0.173
Radiological finding				
X-ray				
Trachitis	49	49	0	0.005
Pneumonia	101	88	13	0.010
Pleural effusion	15	10	5	0.003
Pneumothorax	2	2	0	1.000
Echocardiography (ECHO)				< 0.001
Low pulmonary hypertension	2	0	2	
Intermediate pulmonary hypertension	5	0	5	
High pulmonary hypertension	6	1	5	
Co-infections				
Pertussis -Virus	43	42	1	0.109
Respiratory syncytial virus	23	23	0	0.129
Rhinovirus	10	10	0	0.600
FA	6	5	1	0.439
Parainfluenza	4	4	0	1.000
Adenovirus	0	0	0	1.000
Pertussis -Bacteria	38	31	7	0.043
*Klebsiella pneumoniae*	10	7	3	0.058
*Staphylococcus aureus*	10	9	1	1.000
*Haemophilus influenzae*	7	5	2	0.122
*Streptococcus pneumoniae*	4	3	1	0.318
*Pseudomonas aeruginosa*	3	3	0	1.000
*Escherichia coli*	2	2	0	1.000
*Acinetobacter baumannii*	1	1	0	1.000
*Moraxella catarrhalis*	1	1	0	1.000
Pertussis—*Mycoplasma pneumoniae*	14	11	3	0.117
Pertussis—*Chlamydia pneumoniae*	5	4	1	0.381

### Treatment and outcome

Of the 144 patients with *B. pertussis* infection, 38 (26.4%, 38/144) patients were admitted to the PICU (19.4%, 28/144) and NICU (6.9%, 10/144); 13 (9.0%, 13/144) patients died, with 8 (5.6%, 8/144) patients doing so in the PICU and 5 (3.5%, 5/144) in the NICU. The mortality of severe pertussis in the PICU/NICU was 34.2% (13/38), with patients younger than 6 weeks accounting for 76.9% (10/13) of the deaths. Additionally, 22.2% (32/144) patients needed oxygen and 24.3% (35/144) patients needed assisted ventilation, including mechanical ventilation in 20.1% (29/144) and non-invasive ventilation in 4.2% (6/144), of whom 10.4% (15/144) needed assisted ventilation for > 7 days. The median length of the PICU or NICU stay was 8.5 days (IQR 2.0–18.0 days). The median length of hospitalization was 13 days (IQR 8.0–19.0 days). Table 4 shows the treatment of patients as follows: 97.2% (140/144) patients received macrolide treatments. Most of the patients (86.8%, 125/144) received macrolide treatments > 7 days from the onset of symptoms. Of the patients, 24.3% (35/144) received immunoglobulin therapy. Additionally, 7.6% (11/144) of patients underwent exchange blood transfusion, 4.2% (6/144) of patients underwent CRRT, and 2.8% (4/144) of patients received inhalation of NO.

**Table 4:** Treatments of 144 hospitalized children with pertussis

Characteristics	Total	Survivors	Deaths	P-value
	N = 144	N = 131	N = 13	
	Number	Number	Number	
Macrolide treatments				
No use	4	3	1	0.001
< 7 days after the onset of symptoms	15	10	5	
7–14 days after the onset of symptoms	64	58	6	
> 14 days after the onset of symptoms	61	60	1	
Azithromycin	118	108	10	0.704
Erythromycin	28	24	4	0.281
Corticosteroid therapy	24	17	7	0.001
Immunoglobulin therapy	35	31	4	0.518
Respiratory support treatments				
Oxygen	32	32	0	0.038
Non-invasive ventilation	6	5	1	0.439
Need for conventional mechanical ventilation	18	9	9	< 0.001
Need for high-frequency ventilation	11	8	3	0.062
The length of Mechanical ventilation > 7 days	15	13	2	0.627
Life support treatments				
Exchange blood transfusion	11	8	3	0.062
CRRT	6	3	3	0.010
Inhalation of NO	4	0	4	< 0.001
ECMO	2	1	1	0.173

### Mortality risk factors in hospitalized children with severe pertussis

Univariate analysis of mortality risk factors in hospitalized children with severe pertussis are shown in Tables 1, 2, 3 and 4. Table 1 shows the demographics and baseline characteristics of the patients (all P < 0.05). The host factors associated with death were younger age and lower birth weight. Table 2 presents the clinical characteristics and complications associated with death, which were cyanosis, oxygen saturation < 90%, PH, septic shock and so on (all P < 0.05). Table 3 shows that the factors related to the death of patients and laboratory and radiological findings, and pathogenesis include WBC > 70.0 × 10^9^/L, absolute lymphocyte count > 20 × 10^9^/L, more severe infiltrations on chest radiography, etc. (all P < 0.05). Table 4 shows that the factors related to the death of patients and treatment include the need for mechanical ventilation, inhalation of NO, etc. (all P < 0.05).

Multivariate analysis of mortality risk factors in hospitalized patients with severe pertussis are shown in Table 5. On the multivariate analysis, the independent risk factors for death were WBC > 70.0 × 10^9^/L (odds ratio [OR], 230.66; 95% confidence interval [CI], 5.16–10,319.09 P = 0.005) and PH (OR, 323.29; 95% CI 16.01–6529.42; P < 0.001).

**Table 5:** Risk Factors for deaths of 144 hospitalized children with pertussis on Multivariate Analysis

Variables	β	P	OR	OR 95% CI	
				Lower	Upper
Pulmonary hypertension	**5.7785**	** < 0.001**	**323.29**	**16.01**	**6529.42**
WBC		0.03			
WBC 30.0–50.0 × 10^9^/L	0.6555	0.735	1.93	0.04	85.15
WBC 50.0–70.0 × 10^9^/L	2.3441	0.195	10.42	0.3	361.22
WBC > 70.0 × 10^9^/L	**5.4410**	**0.005**	**230.66**	**5.16**	**10,319.09**
WBC < 30.0 × 10^9^/L				Reference = 1	

The bold means significance level at P < 0.05

## Discussion

According to the previous literature, higher rates of pertussis, hospitalizations, complications, and mortality were in infants than any other age group [10]. In the present study, pertussis mainly occurred in children aged < 6 months, especially those aged < 3 months, which is in line with previous reports [10, 11], and most of the patients were unvaccinated with regard to diphtheria-tetanus-pertussis (DTP). The outbreaks of pertussis have been reported periodically every two to five years [12]. In our study, there was an obvious increase in the numbers of patients and deaths in 2018–2019. This may be due to the increasing laboratory tests of pertussis in our hospital in the last two years. The mortality of severe pertussis has been reported to be very high in developed countries, ranging from 19.7 to 31% [13, 14]. The mortality of pertussis was 9.0% in our study, and the mortality of severe pertussis in the PICU/NICU was 34.2%. The mortality of severe pertussis might be lower than that in reality because of the limitations of the laboratory tests and the inadequate recognition of this disease in its early stage. Some pertussis patients who died were not tested for *B. pertussis* and might be misdiagnosed. Previous reports have indicated that the mortality of pertussis in infants younger than 6 weeks was much higher [4]. In our study, 56.9% of patients aged < 3 months died, 92.3% were younger than 3 months and 76.9% were younger than 6 weeks. This means that these children were not protected by the vaccine, because the recommended schedule for DTP vaccination is from 3 months of age in our country. In 2015, the WHO recommended that the primary DTP vaccination should be given at 6 weeks, at least not later than 2 months [3]. From the results of our research, we also recommend the first dose of DTP should advance to the age of 2 months or even 6 weeks in China.

Based on other publications, younger age, lower birth weight and younger gestational age were the risk factors for death [15, 16], and age < 3 months and underlying comorbid conditions were the independent risk factors for death [17]. In our study, we identified that host factors such as younger age and lower birth weight were significantly associated with the death of patients with severe pertussis. However, it was an unexpected finding that younger age was not found to be an independent risk factor, likely because most of the pertussis patients in our study aged < 6 months, especially those aged < 3 months, would affect the data analyses, leading to bias om the result. On the other hand, we identified that clinical manifestations, such as fever ≥ 38.5℃, cough ≥ 14 days, cyanosis, shortness of breath, fatigue with feeding, seizures, altered sensorium, oxygen saturation < 90%, increased respiratory rate, increased heart rate, crackles, and capillary filling time(CRT) > 2 s were significantly associated with the death of severe pertussis patients. These factors would be some important hints to help paediatric doctors recognize severe pertussis patients in the early stage of the illness.

Severe pertussis was accompanied by a wide range of complications, such as pneumonia, pneumothorax, PH, haemorrhage from the gastrointestinal or respiratory tract, toxic encephalopathy, and septic shock being the most common reported [18–20]. In our study, the presence of pneumonia, pleural effusion, pneumothorax, respiratory failure, ARDS, pneumorrhagia, PH, heart failure, sepsis, toxic encephalopathy, and acute renal failure were associated with death. Pneumonia was the most common complication in severe pertussis and was significantly associated with death [18, 20]. All our dead cases presented with more severe pneumonia effusion, which was more likely to develop to ARDS or pneumorrhagia. ARDS and pneumorrhagia were associated with death from severe pertussis in our univariable analysis, which coincides with prior evidence. In our study, bacterial coinfections were detected in 26.4% besides *B. pertussis* and 31.9% patients had fever. This might indicate secondary bacterial infections, mainly pneumonia, pleural effusion, or even septic shock. Beside macrolide antibiotics, some severe patients in our study needed other antibiotics such as cefoperazone, meropenem, and vancomycin et in the ICU.

PH was present in only 12 patients in our study, but it was a strong predictor of death, as well as an independent risk factor for death. A previous study reported refractory PH in fatal pertussis, which is often associated with hyperleukocytosis [21]. In our study, the leucocytosis in one of patient who died exceeded 100 × 10^9^/L (103.23 × 10^9^/L). We identified that leucocytosis > 70.0 × 10^9^/L and absolute lymphocyte count > 20 × 10^9^/L were also significantly associated with the deaths of patients with severe pertussis, and leucocytosis (> 70.0 × 10^9^/L) was an independent risk factor for death. The mechanism of PH and hyperleukocytosis occurred in severe pertussis due to pertussis toxin (PT) [18]. PT can affect cellular signalling and promote leucocytosis with lymphocytosis, which can result in a hyperviscosity syndrome [22]. Previous reports have shown that luminal aggregates of leucocytes have been observed in pulmonary arterioles, veins and lymphatics of post-mortem lung tissue from infants who died from pertussis [23]. Abnormal leucocyte aggregation in the lungs can diminish blood flow by increasing vascular resistance, which may lead to PH [24]. In addition, pertussis pneumonia may trigger hypoxia, acidosis, acute pulmonary vasoconstriction, microcirculation disturbances, and clotting dysfunction. All these compounded effects produce markedly elevated pressures in the vasculature of the lung that could trigger irreversible PH [24, 25]. Meanwhile, PT is a known inhibitor of G-proteins, which are cardioprotective [26]. PT could alter the vagal control of the heart rate and respiratory rate through the regulation of G-proteins [18, 27]. The rapid increase in heart and respiratory rates, which were associated with death in our cases. Future prospective studies are needed to assess the mechanism of severe pertussis.

The management of severe pertussis is extremely challenging, especially when accompanied by PH and hyperleukocytosis. Most of the patients received macrolide treatments, and some severe patients received immunoglobulin therapy. Previous literature has reported that using inhalation of NO to treat neonatal PH can significantly shorten treatment time and reduce mortality [28]. However, in our study, PH did not improve in four infants who received inhalation of NO. The traditional approach to reduce pulmonary vascular resistance, such as inhalation of NO, may fail because of hyperviscosity and vascular obstruction [22]. Our data suggest that inhalation of NO may not be useful in pertussis-related PH but more samples are needed to confirm this hypothesis. Exchange blood transfusion, which is frequently conducted in the NICU, was first published in a patient with severe pertussis [29]; thereafter, exchange blood transfusion has been reported in multiple case series and case reports of severe pertussis to reduce the level of the total leucocyte count [13, 18, 22]. In our study, 11 patients with severe pertussis in the PICU/NICU underwent exchange blood transfusion, eight patients survived and three died. Exchange blood transfusion can reduce the levels of the leucocytosis and thrombosis in patients with severe pertussis, thereby improving the severity of PH. In addition, two patients who underwent exchange blood transfusion in the early stages of disease also underwent ECMO therapy when exchange blood transfusion did not seem to work. One patient survived, while the other died because of refractory heart failure and septic shock. The use of ECMO in severe pertussis has been reported with some success in small series [18, 22, 30], with a survival rate of only 30%, and the mortality remains higher than that for other indications for ECMO [30]. Further and larger prospective studies are urgently needed to confirm the critical time of exchange blood transfusion and to define the optimal use of ECMO in severe pertussis in order to decrease its mortality.

The study limitation was lacking the attentions about the macrolide resistance of pertussis. The previous lectures showed a strikingly high rate of macrolide resistance in B. pertussis in China (85–91.9%), especially in erythromycin [31, 32]. The macrolide resistance might be one reason for the high frequency of severe pertussis in our hospitalized patients. But B. pertussis culture and drug sensitivity were not tested in our hospital. Further investigations should be undergoing to test the macrolide resistant B. pertussis genes which may help to reduce the high frequency of severe pertussis and mortality in these hospitalized pertussis patients in China.

## Conclusions

In our study, severe pertussis mainly occurred in children aged < 3 months, and most of the patients were unvaccinated with regard to pertussis. The mortality of severe pertussis was 34.2%, with the severe patients younger than 6 weeks accounting for the majority of deaths (76.9%). We recommend the first dose of DTP should advance to the age of 2 months or even 6 weeks. The presence of WBC > 70.0 × 10^9^/L and PH were the prognostic independent variables associated with death. Our data also suggest that inhalation of NO may not be useful in pertussis-related PH.

## Abbreviations

ARDS: Acute respiratory distress syndrome
CRRT: Continuous renal replacement therapy
CRT: Capillary filling time
DTP: Diphtheria-tetanus-pertussis
ECHO: Echocardiography
ECMO: Extracorporeal membrane oxygenation
ERS: European respiratory society
ESC: European Society of Cardiology
HRCT: High-resolution computed tomography
IQR: Interquartile range
NICU: Neonatal Intensive Care Unit
PCR: Polymerase chain reaction
PH: Pulmonary hypertension
PICU: Paediatric Intensive Care Unit
PT: Pertussis toxin
NO: Nitric oxide
